# A value-added multistage utilization process for the gradient-recovery tin, iron and preparing composite phase change materials (C-PCMs) from tailings

**DOI:** 10.1038/s41598-019-50536-y

**Published:** 2019-10-01

**Authors:** Zijian Su, Yikang Tu, Xijun Chen, Yuanbo Zhang, Benlai Han, Corby Anderson, Tao Jiang

**Affiliations:** 1 School of Minerals Processing and Bioengineering, Central South University, Changsha, 410083 China; 2 Department of Metallurgical and Materials Engineering, Colorado School of Mines, Golden, 80401 United States

**Keywords:** Environmental chemistry, Environmental impact

## Abstract

Tin-, iron-bearing tailing is a typically hazardous solid waste in China, which contains plenty of valuable tin, iron elements and is not utilized effectively. In this study, a multistage utilization process was put forward to get the utmost out of the valuable elements (tin and iron) from the tailings, and a gradient-recovery method with three procedures was demonstrated: (1) An activated roasting followed by magnetic separation process was conducted under CO-CO_2_ atmosphere, tin and iron were efficiently separated during magnetic separation process, and 90.8 wt% iron was enriched in magnetic materials while tin entered into non-magnetic materials; (2) The tin-enriched non-magnetic materials were briquetted with CaCl_2_ and anthracite and roasted, then tin-rich dusts were collected during the chloridizing roasting process; (3) The roasted briquettes were infiltrated in melting NaNO_3_ to prepare NaNO_3_/C-PCMs by a infiltration method. Three kinds of products were obtained from the tailings by the novel process: magnetic concentrates containing 64.53 wt.% TFe, tin-rich dusts containg 52.4 wt.% TSn and NaNO_3_/C-PCMs for high temperature heat storage. Such a comprehensive and clean utilization method for tin-, iron-bearing tailings produced no secondary hazardous solid wastes, and had great potential for practical application.

## Introduction

Tin is a crucial cog in the global economy as its use in electronic solder industry, since the third revolution of science and technology^1–4^. Cassiterite (SnO_2_) is the only natural mineral from which tin metal can be economically extracted, and more than 90% tin was obtained from the processes of physical mineral processing and carbothermic reduction smelting^4,5^. The average tin content of primary tin ores was only about 0.2–1.0 wt.%, while the minimum tin grade of cassiterite concentrates for smelting was above 40.0 wt.%, and the recovery of tin was as low as 10–20 wt.% during the long process of multistage mineral processing^5^. Therefore, a large fraction of tin-bearing tailings with 0.1–1.0 wt.% tin content was produced during the beneficiation processes and discharged as solid waste. According to incomplete statistics, about more than 10 million tons of tin-bearing tailings are produced each year in China, and the total amount of tin-bearing tailings is larger than 500 million tons in the world^6^. The tailings not only occupy precious land and cause environmental pollution, but also give rise to security risks^6–8^.

Tin is classified into siderophile element according to Goldschmidt geochemical classification of the elements. Hence, tin-, iron-bearing minerals are closely associated and fine-grained during the geological mineralization process^9,10^. Nevertheless, iron is a lower-value element compared to tin, and iron-bearing minerals are always discarded into the tailings dam. Moreover, iron has adverse effects on the tin recovery during the physical mineral beneficiation process and tin smelting process, which brings difficulty for utilizing tin-bearing tailings. Based on the type of iron-bearing minerals, the tin-bearing tailings were classified into hematite-limonite type and magnetite type tailings, and previous investigations showed that both types were difficult to utilize^4,5^.

The high-grade tin resources have been almost exhausted in recent years, especially in China, so the secondary tin resources of tin-bearing tailings have been paid much attention^1,4,5^. Advanced gravity concentration and flotation methods are applied to recover tin from these tailings, but the complex disseminated relationship of tin and iron minerals can’t be changed during these physical dressing processes^11,12^. In addition, Fe^3+^ ions were easily dissociated from the iron minerals during the mineralization process, and adsorbed on the surfaces of cassiterite particles, which restrained the effectiveness of flotation reagents^4^. Fuming processes were practised to recover tin efficiently from low-grade tin-bearing materials, but the iron oxides were easily reduced to FeO and generated low-melting-point slags. Moreover, those iron oxides were impossible to recover, so the iron-bearing slags became secondary hazardous solid wastes^13,14^. Nowadays, the traditional processes were mainly focused on Fe or Sn extraction from tin-bearing tailings, which had low efficiency and caused secondary contamination.

Slags generated from the pyrometallurgical industry are regarded as hazardous solid wastes, and they are also deemed to be with low economic value and difficult to utilize^15–18^. However, series of natural minerals and slags were commonly used to prepare composite phase change materials (C-PCMs) for thermal energy storage, which provided new insights to utilize metallurgical slags through environment-friendly and high-value-added processes^19–22^. Natural clay minerals (kaolin, diatomite, sepiolite, bentonite, perlite, etc) have porous structure and considerable specific surface area, thus, those minerals were always with excellent adsorbability and suitable for preparing low-temperature C-PCMs (~100 °C)^23–26^. Industrial solid wastes such as fly ash (FA) and blast furnace slags (BFS) were obtained from high-temperature processes, which showed excellent thermostability and corrosion resistibility. Then, novel routes to utilize FA and BFS for preparing high-temperature C-PCMs (higher than 300 °C) were proposed, and PCMs (NaNO_3_, Al, Na_2_SO_4_, etc) with different operating temperatures were selected^22^.

Considering the present lower utilization of the tin-bearing tailings and urgent need of tin resources, it is imperative to develop a comprehensive utilization route for these tin-bearing tailings. Our group has paid substantial attention to develop effective method to utilize the tailings. In this study, a value-added multistage utilization process for the gradient-recovery Sn, Fe and preparing C-PCMs from tailings was proposed. High quality magnetite concentrates, tin-bearing dusts and NaNO_3_/C-PCMs were produced from the tailings, and no secondary hazards were produce during the whole process.

## Materials and Method

### Materials

Two typical tin-bearing tailings (tailing A and tailing B) used in this study were taken from Yunnan province, China. The major chemical compositions of them were determined by XRF, the total Fe and Sn content were detected by ICP-AES, and the results are shown in Table 1. The tin content of the tailings was respectively 0.792 wt.% and 0.358 wt.%, while the iron content were 40.15 wt.% and 36.00 wt.%, and both of iron and tin in the tailings were with great value. The sieve analysis results of the tailings indicated that the granularity of the tailings below 0.074 mm was found as 78.26 wt.% and 74.26 wt.%, respectively.

**Table 1:** Chemical compositions of the tin-, iron-bearing tailings.

Components (wt.%)	TFe	FeO^a^	SiO_2_	Al_2_O_3_	CaO	MgO	Sn	S	LOI
Tailing A	40.15	1.23	1.50	1.20	13.62	5.42	0.792	0.21	13.32
Tailing B	36.00	15.32	18.85	4.30	10.46	2.01	0.358	0.10	4.37

^a^FeO content is analyzed by a chemical method.

**Figure 1:**
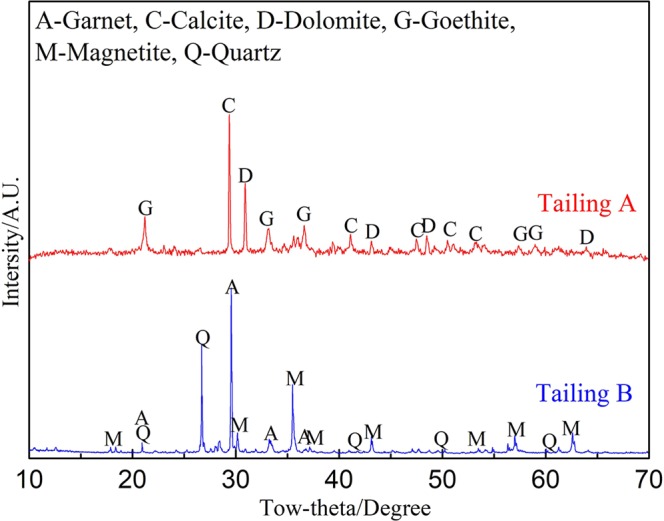
XRD pattern of the tailings.

The XRD pattern of the tailings are shown in Fig. 1. Figure 1 indicated that the major mineral compositions of Tailing A were goethite, calcite and dolomite, while magnetite, quartz, garnets (mainly andradite (Ca_3_Fe_2_(SiO_4_)_3_) and grossularite (Ca_3_Al_2_(SiO_4_)_3_)) were the main phases in Tailing B. Based on the types of iron-bearing minerals, the tailings were typical hematite-limonite type (Tailing A) and magnetite type (Tailing B).

**Figure 2:**
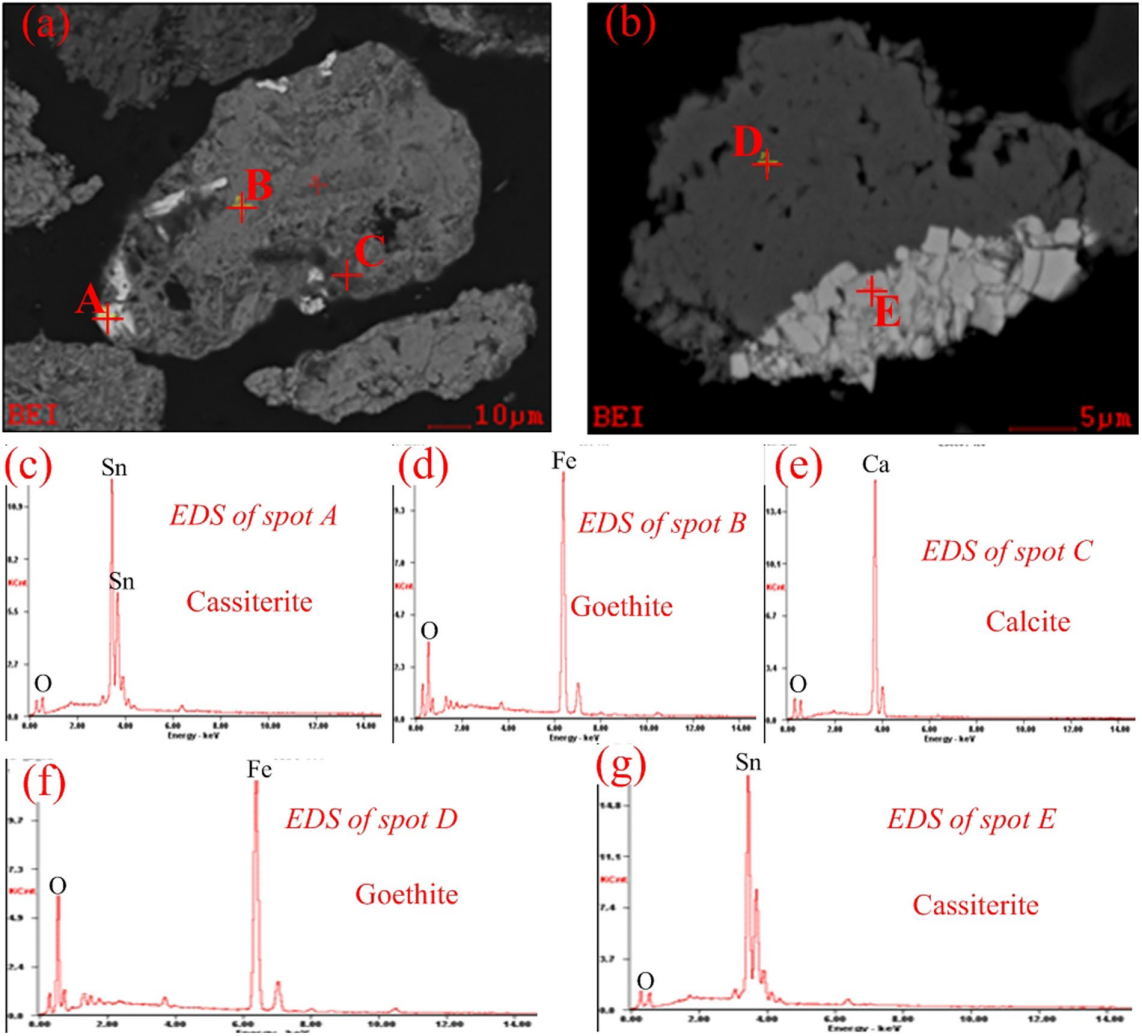
SEM-EDS images of the Tailing A (hematite-limonite type).

**Figure 3:**
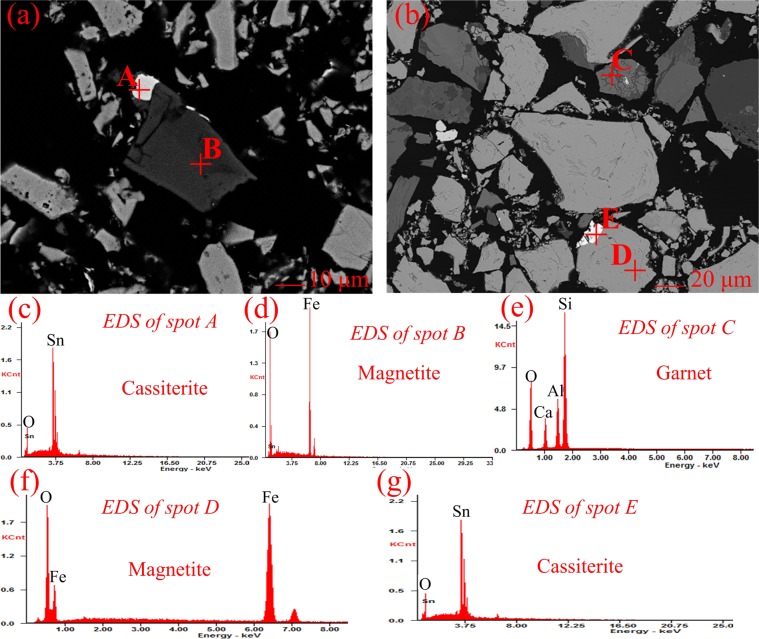
SEM-EDS images of the Tailing B (magnetite type).

The microstructure of the tailings were observed by SEM-EDS, and the results are shown in Figs 2 and 3. As observed from Figs 2 and 3, the particle size of cassiterite minerals was less than 10 μm, and they always existed as individual particles or were closely embedded in iron-bearing minerals (Point A and Point E in Fig. 2, Point A and Point E in Fig. 3). Based on the previous researches, ultrafine particle (<10 μm) and unliberated cassiterite minerals are almost unrecoverable by conventional physical separation methods^4,5^.

The tin content in the tailings was too low to determine the phase compositions by XRD and SEM-EDS, hence, chemical phase analysis was used to make sure the phases of tin in the tailings in this study^27,28^. The results in Table 2 showed that the distribution ratio of tin in cassiterite existed as cassiterite in the tailings were 98.6 wt.% and 68.4 wt.%, respectively. Previous studies indicated that part of tin existed as lattice Sn^4+^ in magnetite, which were more difficult to recover^4,29^.

**Table 2:** Phase compositions of tin in the tailings.

	Tin existed as cassiterite		Acid-soluble tin	
	Content (wt.%)	Distribution ratio (wt.%)	Content (wt.%)	Distribution ratio (wt.%)
Tailing A	0.781	98.6	0.011	1.4
Tailing B	0.245	68.4	0.113	31.6

### Methods

**Figure 4:**
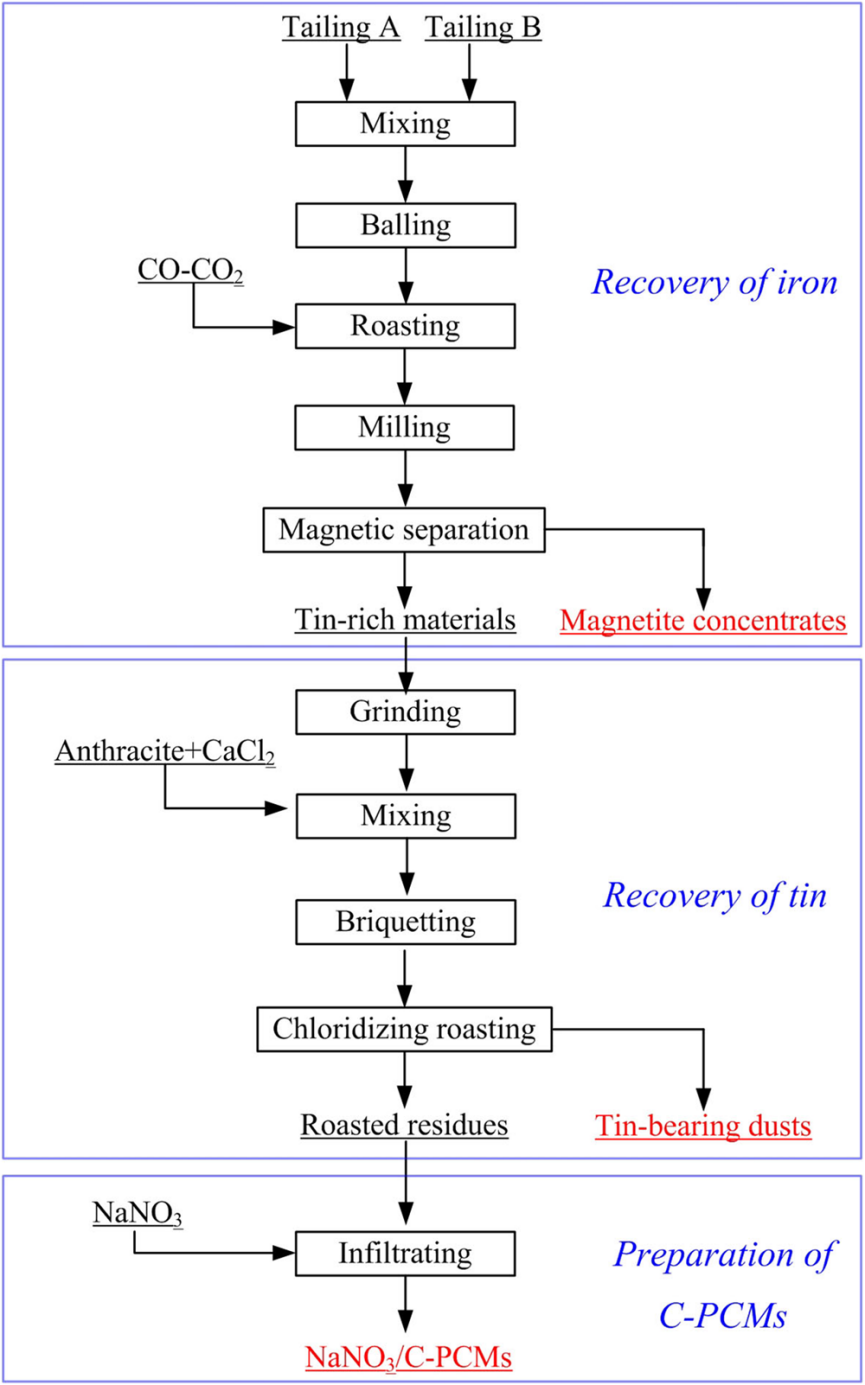
The experimental flowsheet.

The experimental flowsheet is shown in Fig. 4. All the roasting tests were conducted in a horizontal resistance furnace as presented in our previous studies^5^. As shown in Fig. 4, both of Sn and Fe were recovered gradiently, and NaNO_3_/C-PCMs were prepared from the tailings by the following three steps.

#### Recovery of iron

Two tailings were mixed as mass ratio of 1:1, and the mixed samples were balled into 8–10 mm green pellets in a disk pelletizer. After dried, the pellets were weighed and roasted in the horizontal furnace. The total flow rate of the CO-CO_2_ mixed gas was fixed at 4.0 L/min. After roasted at given temperatures for a period, the pellets were subjected to wet-grinding in a ball mill (XMQ Φ240*90) for 10 min, and the grinding concentration was fixed at 50 wt.%. After wet-grinding, the magnetic separation was conducted in a magnetic separator (Model: XCGS-73, Davis magnetic tube) with a magnetic field intensity of 0.10T. The grinding and magnetic separation parameters were chosen based on our previous study^5^. Finally, the magnetite concentrates were obtained and most of tin was enriched in non-magnetic materials (tin-rich materials).

#### Recovery of tin

The non-magnetic tin-rich materials were ground to 100 wt.% less than 0.074 mm, and then mixed with a certain amount of CaCl_2_ and anthracite (the fixed carbon content was 82.3 wt.%, and the granularity below 0.037 mm was 100 wt.%). Next, the mixture was pressed into cylindrical green briquettes with diameter of 5 mm and height of 5 mm under a pressure of 20 MPa for 3 min. After that, the green briquettes were dried and then roasted in the horizontal furnace. The total flow rate of the N_2_ gas was fixed at 4.0 L/min. Finally, the tin-bearing dust was collected from the offgas.

#### Preparation of C-PCMs

After the chloridizing roasting process, the temperature of the horizontal furnace was cooled to 350 °C, then the briquettes were infiltrated in melting NaNO_3_ at 350 °C for 2 h^22^. Finally, the NaNO_3_/C-PCMs were prepared and cooled to room temperature. Thermal cycle tests were carried out in the muffle furnace and the C-PCMs were maintained at a temperature of 350 °C for 2 h before dropping to 50 °C during each thermal cycle.

### Characterization

The mineral constituents of the roasted samples were identified by X-ray diffraction (XRD, D/max 2550PC, Japan Rigaku Co., Ltd) with the step of 0.005° at 10° min^−1^ in ranging from 10° to 80°, then XRD results was analyzed using Jade.6.0. The major chemical compositions were determined by XRF (X-ray Fluorescence, Axios mAX), and the content of Fe and Sn content were detected by ICP-AES (Inductively Coupled Plasma-Atomic Emission Spectrometry, IRIS Intrepid II XSP). The morphological change of the sample was detected using an environmental scanning electron microscope (ESEM; FEI QUANTA 200; FEI, Eindhoven, The Netherlands) equipped with an EDAX energy dispersive x-ray spectroscopy (EDS) detector (EDAX Inc., Mahwah, NJ). The latent heat and the phase-transition temperature of the PCMs and the prepared C-PCMs were characterized by differential scanning calorimeter (DSC, NETZSCH STA 449F3) in an argon atmosphere at a heating rate of 5 °C/min. In this study, thermal cycling test was carried out to determine the change in thermal properties of C-PCMs. Thermal reliability of the prepared C-PCMs was tested by 50 thermal cycles of heating and cooling between 50 °C and 350 °C at a rate of 5 °C/min. Thermal cycle test was carried out in the muffle furnace and the C-PCMs were maintained at a temperature of 350 °C for 2 h before dropping to 50 °C during each thermal cycle^22^. The heat transfer property of the C-PCMs was examined by a laser thermal conductivity meter (NETZSCH LFA 457) in an air atmosphere at a heating rate of 5 K/min and the sample used was pressed into wafer with a diameter of 10 mm and thickness of 2 mm^22^. The thermal diffusion coefficient a (m^2^·s^−1^) using the laser thermal conductivity meter and the specific heat capacity Cp (J·kg^−1^·K^−1^) was tested by Thermal Analyzer. Then the thermal conductivity coefficient of λ (W/m·K) was calculated as: λ = Cp × α × ρ^22^.

### Thermodynamic analysis

The main objective of the thermodynamic analysis was to predict the effect of the temperature and CO content on the reduction of iron and tin-bearing materials, and Factsage 7.2 software was used. The calculations were based on the minimization of the Gibbs free energy of the system under isothermal and isobaric conditions.

## Results and Discussion

### Recovery of iron

Based on our previous studies, tin-bearing minerals in tailings were fine-grained cassiterite and lattice Sn^4+^ in magnetite particles, and both types of tin were very hard to separate and recover by traditional processes. It was found that cassiterite and lattice Sn^4+^ in magnetite could react with CaO to form CaSnO_3_/Ca_2_SnO_4_ as Eqs (1) and (2)^29–31^.

1 $${\rm{CaO}}+{{\rm{SnO}}}_{2}\to {{\rm{CaSnO}}}_{3}/{{\rm{Ca}}}_{2}{{\rm{SnO}}}_{4}$$

2 $${{\rm{Fe}}}_{3-{\rm{x}}}{{\rm{Sn}}}_{{\rm{x}}}{{\rm{O}}}_{4}+{\rm{CaO}}\to {{\rm{Fe}}}_{3}{{\rm{O}}}_{4}+{{\rm{CaSnO}}}_{3}/{{\rm{Ca}}}_{2}{{\rm{SnO}}}_{4}$$

CaSnO_3_/Ca_2_SnO_4_ were nonmagnetic substances while Fe_3_O_4_ was magnetic, which made it possible to separate iron and tin effectively during the following magnetic separation process. Then, magnetite concentrates with very low tin content were produced, and most of tin were enriched in the non-magnetic materials.

As observed form Figs 1–3, the calcite and dolomite in Tailing A were easily decomposed under roasting conditions, which could provide CaO components and promoted the separate of tin and iron^4^. However, Ca-bearing minerals in Tailing B were mainly garnets, which have high thermostability and difficult to decompose to CaO during the roasting process. So, extra additives of CaO must be added during the calcified roasting followed by magnetic separation process^29^. Nevertheless, in this study, Tailing A and Tailing B were mixed first, and the CaO generated from carbonate was enough for the reactions of Eqs (1) and (2), then no extra additives were used in this process.

**Figure 5:**
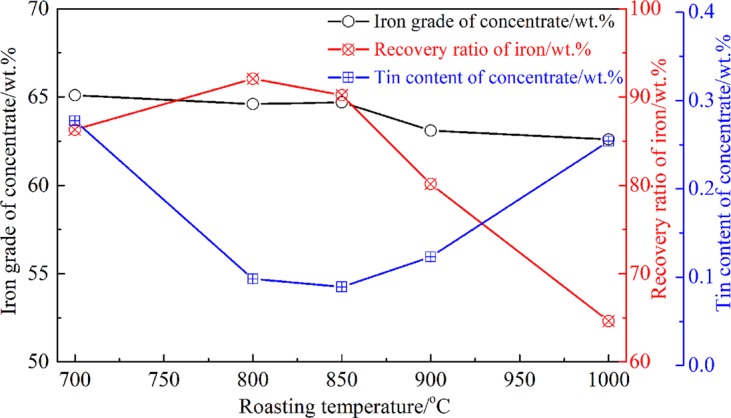
Effect of roasting temperature on the separation of iron and tin from the mixed tailings.

All the experiments were conducted as Fig. 4, and the mixed tailings (Tailing A and Tailing B were mixed as mass ratio of 1:1, with 38.07 wt.% Fe and 0.575 wt.% Sn) were used as the raw materials. Based on our previous studies, the roasting temperature had significant effect on the phase transformation of tin-bearing minnerals and the separation of tin and iron^4,29^. Hence, the effect of roasting temperature on the separation of tin and iron from the mixed tailings were investigated at the temperature range of 700–1000 °C, and the results are shown in Fig. 5. The other roasting conditions were fixed: roasting time of 90 min and roasting atmosphere of 5 vol.% CO/(CO + CO_2_). After that, the roated products were wet-ground to 80 wt.% less than 0.074 mm, and the magnetic separation tests were conducted in the magnetic separator.

Figure 5 demonstrated that it was easy to get high Fe recovery and iron grade of the magnetic materials as the temperature increased from 700 to 850 °C, meanwhile, the tin content in the magnetic materials decreased obviously from 0.268 wt.% to 0.089 wt.%. However, as the roasting temperature increased further to 900 °C and 1000 °C, the iron recovery and iron grade of magnetic materials decreased while the tin content increased slightly, which was attributed to the formation of Ca-Fe-Sn-O and Ca-Fe-O compounds at higher temperature^32,33^.

**Figure 6:**
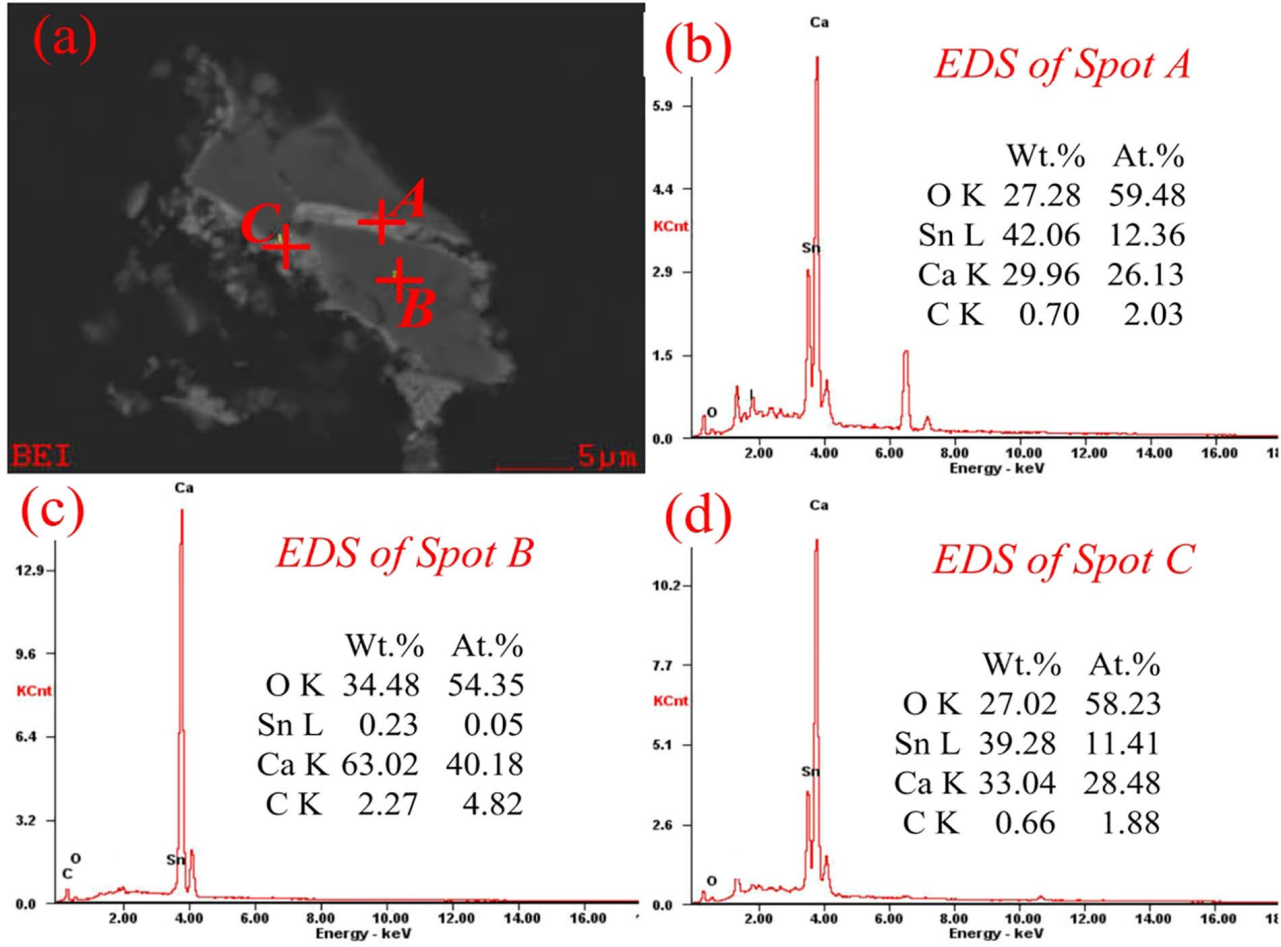
SEM-EDS results of non-magnetic materials obtained under the optimal parameters.

Based on Fig. 5, the results obtained under optimal conditions were listed in Table 3. A magnetic concentrate containing 64.53 wt.% TFe and 0.089 wt.% TSn was obtained, while the tin content of the non-magnetic materials was enriched to 1.226 wt.%. Figure 6 presents SEM-EDS results of the non-magnetic materials, and Ca-Sn-O compounds (Spot A and Spot C in Fig. 6) were found in the outer layer of the Ca-bearing compounds. In addition, the Ca/Sn/O atomic ratio of the Ca-Sn-O compounds (Spot A and Spot C in Fig. 6) was similar to the value of 2:1:4, which confirmed the formation of Ca_2_SnO_4_ as Eqs (1) and (2).

**Table 3:** The results obtained under the optimal conditions.

	Yeild/wt.%	Grade/wt.%		Recovery/wt.%	
		Fe	Sn	Fe	Sn
Raw materials (mixed tailings)	—	38.07	0.575	—	—
Roasted products	92.5	41.16	0.622	100.0	100.0
Magnetite material	53.6	64.53	0.089	90.8	8.3
Non-magnetite material	43.0	8.10	1.226	9.2	91.7

### Recovery of tin

As demonstrated in Section 3.1, the tin grade of non-magnetic materials was enriched to 1.226 wt.%, and most of tin was transformed to Ca_2_SnO_4_. It was known that chlorination volatilization method is the most efficient method to recover tin from middle-grade tin materials. So, CaCl_2_ was chosed as chlorination agents in this study. Firstly, the possible volatilization reactions of Ca_2_SnO_4_ were taken into consideration. Table 4 lists the possible reactions and ∆G^θ^-T equations of Ca_2_SnO_4_-CaCl_2_-CO-CO_2_ system. The values of thermodynamic parameters of the pure substances are obtained from FactSage 7.2.

**Table 4:** ∆G^θ^-T equations of the possible reactions of Ca_2_SnO_4_-CaCl_2_-CO-CO_2_ system.

Eqs.	Reactions	∆G^θ^-T (KJ/mol)
(3)	1/2Ca_2_SnO_4_ + CO = CaO + CO_2_ + Sn	∆G^θ^ = −0.134 T + 106.5
(4)	Ca_2_SnO_4_ + CO = 2CaO + CO_2_ + SnO_(g)_	∆G^θ^ = −0.405 T + 502.2
(5)	Ca_2_SnO_4_ + CaCl_2_ + CO = 3CaO + CO_2_ + SnCl_2(g)_	∆G^θ^ = −0.350 T + 379.0

**Figure 7:**
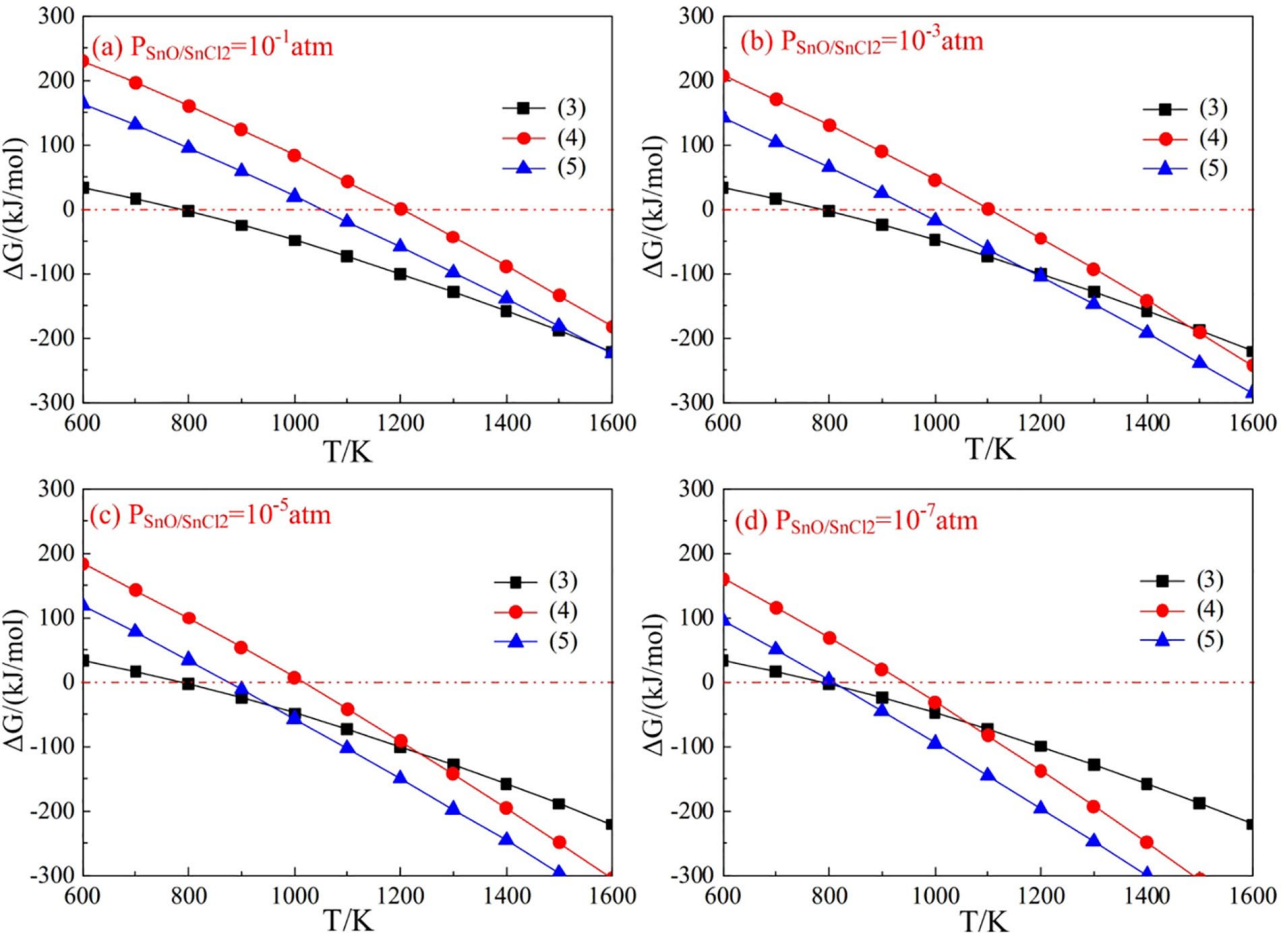
∆Gθ-T plots of Eqs (3)–(5) at various gas partial pressures.

**Figure 8:**
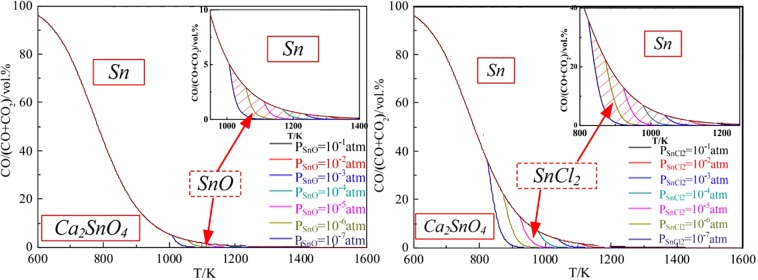
Gas-phase equilibrium diagram of gaseous SnO and SnCl_2_ (p’_SnO_ = 10^−7^–10^−1^ atm).

It was known that both of SnO and SnCl_2_ could volatilize as gaseous phase at a relative lower temperature, hence, the effect of generated gasous products (partial pressure) on the reaction equilibrium were calculated and plotted in Fig. 7^34–37^. Based on the ΔG-T relationship of Eqs (3)–(5), the CO/(CO + CO_2_)-T equilibrium relationship at given SnO/SnCl_2_ partial pressures was calculated, and the gas-phase equilibrium diagram was presented in Fig. 8.

Figure 7 showed that the ∆G value of Eqs (3) and (4) decreased obviously as the decrease of the gas partial pressure, as well as the reaction temperature of the volatilization reactions. In addition, it was observed from Fig. 7 that the ∆G value of Eq. (4) was much higher than that of Eq. (3) at various gas partial pressures, which indicated that Ca_2_SnO_4_ was much easier to be reduced to metallic tin than SnO. However, the ∆G value of Eq. (5) decreased much lower than Eq. (3) when SnCl_2_ gas partial pressure was 10^−5^ and 10^−7^ atm (Fig. 7b,c), then Eq. (6) could be conducted preferentially. The results in Fig. 8 illustrated that the volatilization reactions were controlled by the partial pressure of gaseous SnO/SnCl_2_, and the shadow area of SnO/SnCl_2_ stability zone was increased as the decrease of SnO/SnCl_2_ partial pressure. However, the SnO stability zone (in Fig. 8) was too narrow to control, so it was easier to recover Sn from Ca_2_SnO_4_ in the presence of CaCl_2_.

**Figure 9:**
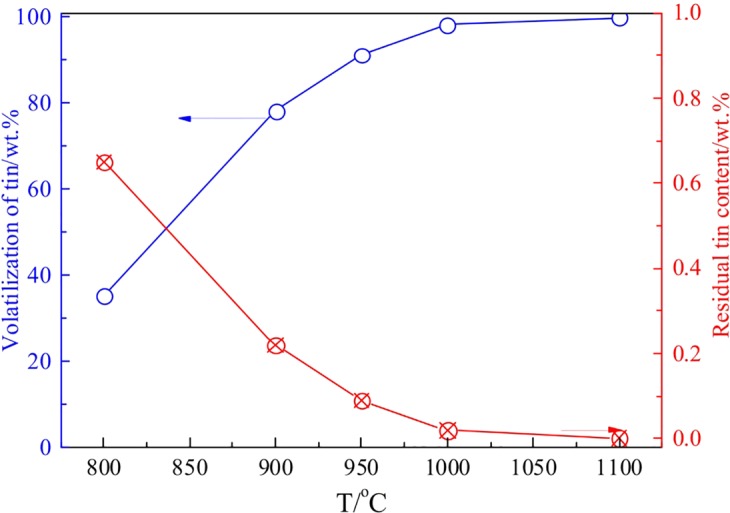
Effect of roasting temperature on the chloridizing volatilization of tin.

The experimental flowsheet of the fuming process was shown in Fig. 4, CaCl_2_ and anthracite were used as chloridizing agent and reducing agent, respectively. The volatilization dusts and roasted residues were collected and analyzed. The effect of roasting temperature on the volatilization of tin was investigated, and the results were shown in Fig. 9. Figure 9 indicated that the temperature had a significant impact on the volatilization ratio of Sn, and the Sn volatilization ratio increased from 32.5wt.% to 99.5 wt.% as the roasting temperature rose up from 800 °C to 1000 °C. Based on the thermodynamic analysis, the saturated vapor pressure of SnCl_2_ increased as the increase of temperature, which promoted the volatilization of tin.

The results obtained under optimal roasting temperature of 1000 °C were listed in Table 5 and Table 6 based on Fig. 9. As shown in Table 5 and Table 6, the Sn content in the dust was enriched to 52.4 wt.% with a recovery of 99.5 wt.%, while the residual Sn content in the roasted briquettes decreased to 0.005 wt.%.

### Preparation of C-PCMs

**Figure 10:**
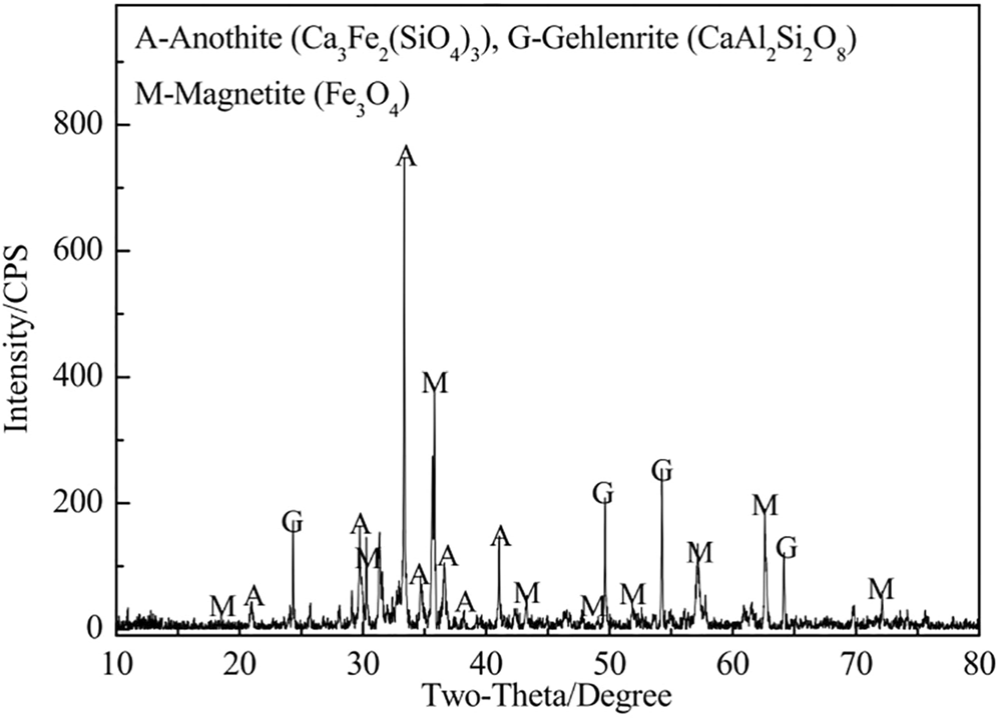
XRD partterns of the chloridizing roasting residues.

According to the results of Section 3.1 and 3.2, both of iron and tin were recovered efficiently, only low-value elements of Si, Ca, Mg and Al were remained in the roasted briquettes as shown in Tables 5 and 6. The XRD pattern of the roasted briquettes shown in Fig. 10 indicated that the main phases were anothite, gethlenrite and magnetite.

**Table 5:** The results obtained under optimal chloridizing volatilization conditions.

	Yeild/wt.%	Content of Sn/wt.%	Recovery of Sn/wt.%
Tin-rich materials	—	1.226	—
Tin-bearing dusts	2.1	52.4	99.5
Roasted briquettes	97.9	0.005	0.5

**Table 6:** Chemical compositions of the chloridizing roasting residues.

Components	TFe	FeO	SiO_2_	Al_2_O_3_	CaO	MgO	Sn	S
wt.%	7.89	2.32	25.52	6.87	32.21	8.25	0.005	0.01

**Figure 11:**
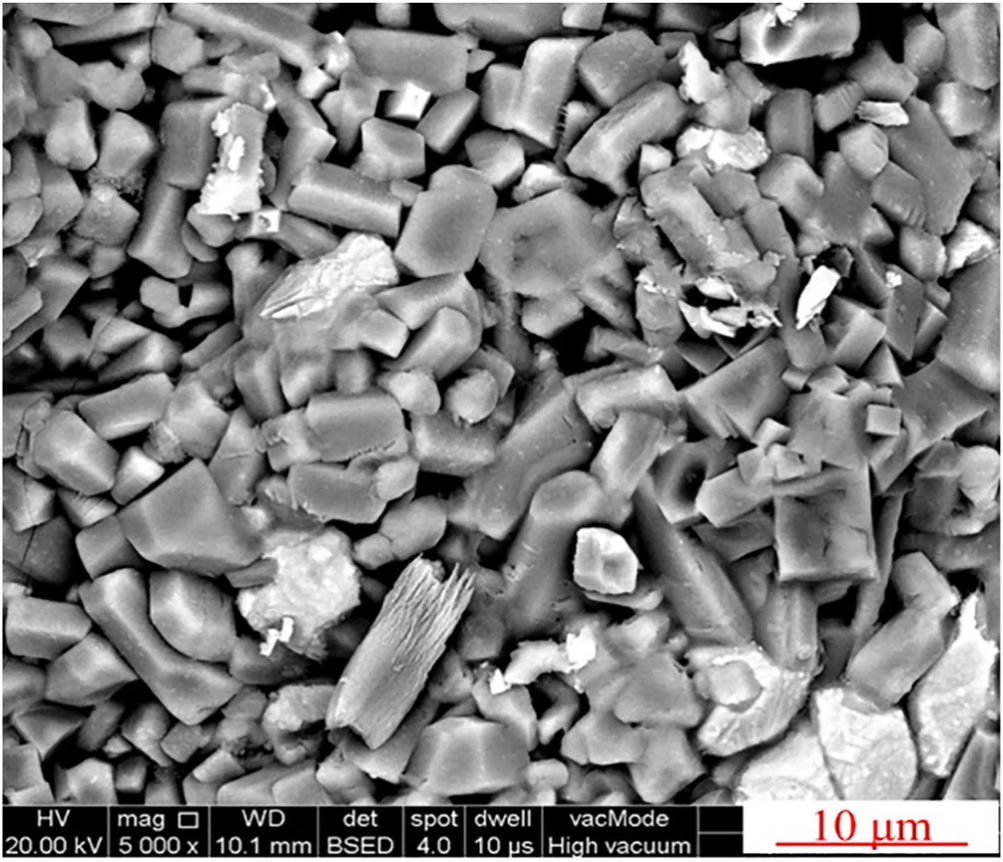
SEM image of the chloridizing roasting residues.

Figure 11 shows the SEM image of the fracture surface of the roasted briquettes, demonstating that the briquettes had more coarse surfaces with a polyporous structure consisting of a large number of irregular micropores in the range of 1–5 micrometer. The chloridizing roasting temperature was much lower than the melting point of the mixtures, so no fusion phase was formed during the roasting process. In addition, the reducing agent of anthracite also played an important role as pore forming materials, so a porous structure was formed after the anthracite was burnt up. The porosity value of the roasted briquettes was determined as 35.2 vol.% by drainage method.

**Figure 12:**
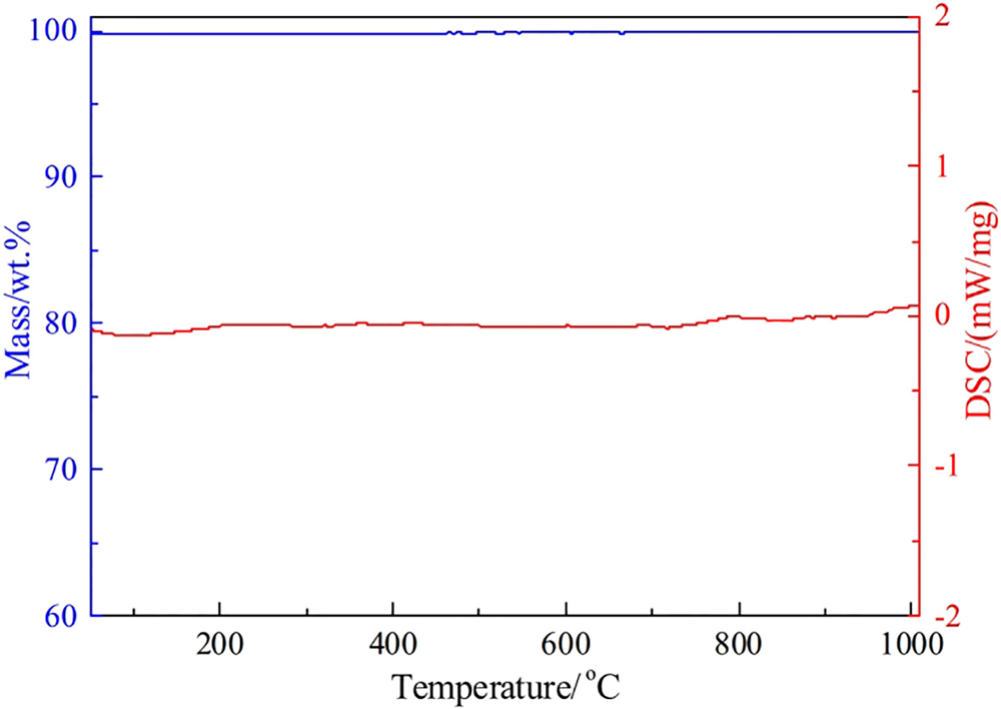
TG-DSC analysis of the chloridizing roasting residues.

The TG-DSC curves of the roasted residues are plotted in Fig. 12. It was seen from Fig. 12 that the mass of the roasted residues almost kept unchanged and no chemical reaction occurred as the temperature rose from room temperature to 1000 °C.

Based on the results in Figs 10–12, it was known that the chloridizing roasted briquettes had a porous structure and excellent thermostability. According to the previous investigations^22^, we proposed to use the roasted briquettes as structural materials for high temperature C-PCMs. It was known that NaNO_3_ was a common PCM with an operation temperature of 300–350 °C. So, we tried to prepare NaNO_3_/C-PCM based on the flowsheet in Fig. 4. The roasted briquettes were first infiltrated in fusion NaNO_3_, then NaNO_3_ was filled into the micropores of the briquettes. Finally, NaNO_3_/C-PCMs were obtained. The mass addition value of the roasted briquettes after infiltration was measured as 61.7 wt.%, indicating that the mass fraction of NaNO_3_ in C-PCMs was 38.2 wt.%.

**Figure 13:**
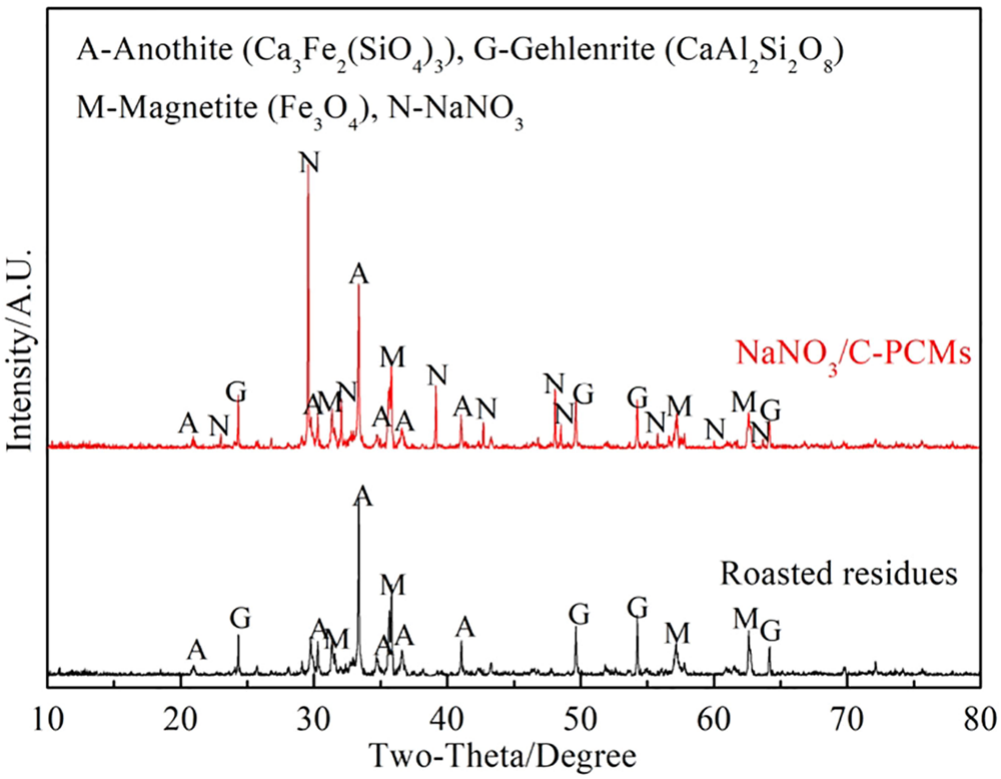
XRD patterns of NaNO3/C-PCMs and roasted residues.

Figure 13 shows the XRD patterns of the prepared NaNO_3_/C-PCMs and original roasted briquettes. In Fig. 13, the diffraction peaks of NaNO_3_, anothite, gethlenrite and magnetite were found, and no newly generated phase was observed, which verified that that NaNO_3_ didn’t react with the structural materials under such conditions. Therefore, the original roasted briquettes and the NaNO_3_ are highly chemically compatible at high temperatures and only physical changes could occur betwwen the two components.

**Figure 14:**
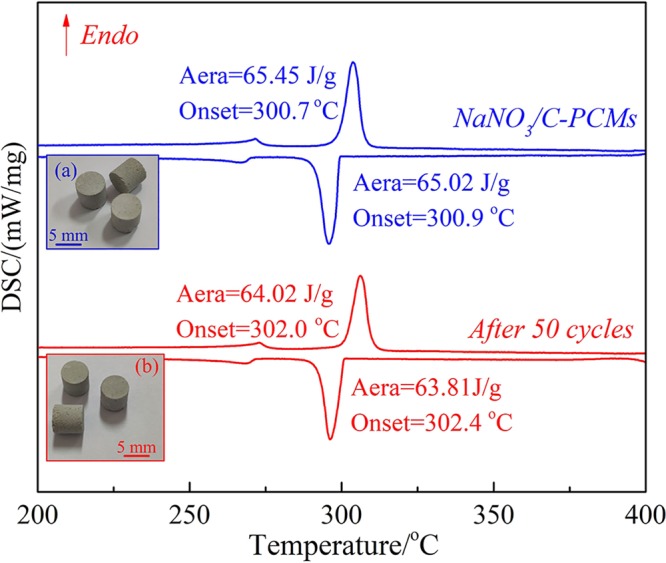
Melting-freezing DSC cures of NaNO_3_/C-PCMs (the insets are the photograph of the NaNO3/C-PCMs before and after 50 cycles).

In order to determine the thermal storage property and thermal stability of the NaNO_3_/C-PCMs, the melting-freezing DSC cures of NaNO_3_/C-PCMs before and after 50 thermal cycles were analyzed, and the results are shown in Fig. 14.

Our previous study indicated that the melting and freezing temperatures for pure NaNO_3_ were measured to be 303.6 °C and 301.1 °C, and the melting and freezing latent heat were 180.23 J/g and 177.96 J/g, respectively^22^. The melting and freezing temperatures of prepared NaNO_3_/C-PCMs in Fig. 14 were measured as 300.7 °C and 300.9 °C, while the melting and freezing latent of NaNO_3_/C-PCMs were 65.45 J/g and 65.02 J/g, which was very close to the theoretical values (NaNO_3_ mass fraction in the C-PCMs was 38.2 wt.%, 180.23 J/g × 38.2 wt.% = 68.85 J/g). In addition, the results illustrated that the super-cooling of the NaNO_3_/C-PCMs was only 0.2 °C compared to 2.5 °C of pure NaNO_3_, which was much favorable to the thermal shock resistance of the C-PCMs^22^. The super-cooling of the prepared NaNO_3_/C-PCMs in this study is even lower than that of C-PCMs using pure compounds as matrix, which was about 2 °C^38^. These results suggest that the prepared NaNO_3_/C-PCMs may be well suited for high-temperature storage applications.

Thermal reliability was the most important property for the C-PCMs, so the DSC curve of the NaNO_3_/C-PCMs was determined after 50 times thermal cycle tests and shown in Fig. 14. The results showed that melting and freezing temperature was 302.0 °C and 302.4 °C, and the melting and freezing latent heat were 64.20 J/g and 63.81 J/g after 50 thermal cycles. Furthermore, it was observed form Fig. 14 that the super-cooling temperature of the C-PCMs after 50 cycles was 0.4 °C, and the phasetransition enthalpy was about 97.8 wt.% of that before the thermal cycles. The thermal reliability of the prepared NaNO_3_/C-PCMs was basically the same as that of other scholars^38,39^. The results revealed that the NaNO_3_/C-PCMs had a good thermal reliability with regard to the changes in the phase change temperature and latent heat. In addition, the appearance and shape of the NaNO_3_/C-PCMs were almost unchanged after 50 thermal cycles as shown in Fig. 14a,b and the products had great apparent compressive strength, which might be suitable for preparing building packing materials^22^.

The heat transfer property is an important character in latent heat energy storage application, which declares the efficiency of heat storage and heat release process. The thermal conductivity coefficient of roasting residues, NaNO_3_ and NaNO_3_/C-PCMs were measured and displayed in Table 7, and the value of NaNO_3_/C-PCMs were 0.70 W/m·K at 25 °C and 0.48 W/m·K at 350 °C. While the values of NaNO_3_ (AR) were 0.54 W/m·K at 25 °C and 0.47 W/m·K at 350 °C. The results in Table 7 indicated that the thermal conductivity coefficient of the prepared composite was reduced with the increase of temperature, and the compact structure between the matrix and NaNO_3_ improved the thermal conductivity of C-PCMs.

**Table 7:** Thermal conductivity of NaNO_3_/C-PCMs under different temperature (W/m·K).

Temp.	25 °C	200 °C	350 °C
Roasting residues	0.53	0.52	0.44
NaNO_3_ (AR)	0.54	0.50	0.47
NaNO_3_/C-PCMs	0.70	0.55	0.48

Moreover, compared to other phase change materials^38–40^, the prepared NaNO_3_/C-PCMs in this study use the cheaper and even harmful solid waste as the main raw materials to convert it into high-value-added heat storage materials. This process not only provides a novel way for the utilization of tin-, iron-bearing tailings, but also has great significance for environmental protection.

**Figure 15:**
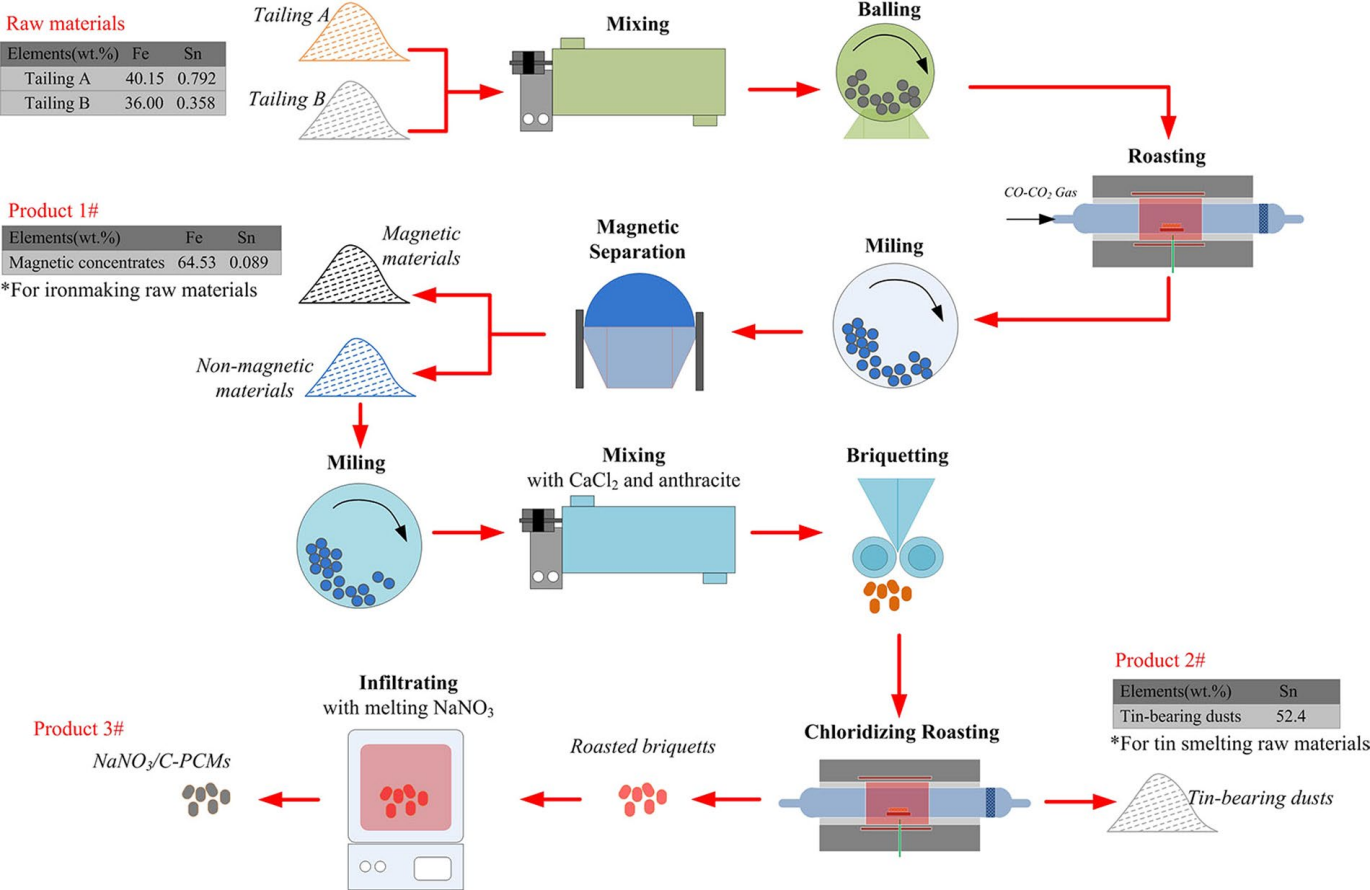
Proposed process for multistage utilization for tin, iron-bearing tailings.

Based on the above researches, we put forward a multistage utilization process for the gradient-recovery Sn, Fe and preparing NaNO_3_/C-PCMs from tin-, iron-bearing tailings, as shown in Fig. 15. Three kinds of products were obtained from the tailings, magnetic concentrates for ironmaking, tin-bearing dusts for tin smelting and NaNO_3_/C-PCMs for thermal energy storage. Both of iron and tin were recovered efficiently with recovery ratio of more than 90 wt.% by the recommended process. It was noteworthy that no secondary hazards was produced during the process, therefore, this study provided a value-added utilization process for the tailings.

## Conclusions

The value-added multistage utilization process for the gradient-recovery Sn, Fe and preparing C-PCMs from tailings was evaluated and the following conclusions were drawn:

Recovery of iron: Both of fine cassiterite and lattice Sn^4+^ in the tailings could react with CaO to form CaSnO_3_/Ca_2_SnO_4_ under roasting conditions, which promoted the separation of tin and iron during magnetic separation process. Magnetic concentrate containing 64.53 wt.% TFe and 0.089 wt.% TSn were obtained, which could be used as raw materials for ironmaking. The tin content in the non-magnetic materials was enriched to 1.226 wt.%.

1. Recovery of tin: Thermodynamic calculation indicated that the tin in calcium stannous was prior to volatilization as SnCl_2(g)_ than SnO_(g)_. A chloridizing volatilization process was conducted for the recovery of tin from the tin-rich non-magnetic materials. Under optimal roasting conditions, the recovery of tin was as high as 99.5 wt.%, and the tin content in the tin-beaing dusts was high to 52.4 wt.%, which met the requirements for tin smelting raw materials.
2. Preparation of C-PCMs: Roasted briquette had a porous structure and excellent thermostability. Melting NaNO_3_ was easily infiltrated into the micropores of the briquette, and NaNO_3_/C-PCMs was prepared by a infiltration method. The melting point and latent heat of the NaNO_3_/C-PCMs were 300.7 °C and 64.20 J/g, respectively. The prepared NaNO_3_/C-PCMs had relatively lower super-cooling of 0.2 °C and very good thermal reliability.
3. This study proposed a value-added multistage utilization process of gradient-recovery Sn, Fe and preparing NaNO_3_/C-PCMs from tin-, iron-bearing tailings, and no secondary hazards were produced during this whole process.
